# Low Persistence of Genetic Rescue Across Generations in the Arctic Fox (*Vulpes lagopus*)

**DOI:** 10.1093/jhered/esab011

**Published:** 2021-03-19

**Authors:** Anna Lotsander, Malin Hasselgren, Malin Larm, Johan Wallén, Anders Angerbjörn, Karin Norén

**Affiliations:** Department of Zoology, Stockholm University, Stockholm, Sweden

**Keywords:** conservation, gene flow, genetic rescue, genetic sweep, inbreeding

## Abstract

Genetic rescue can facilitate the recovery of small and isolated populations suffering from inbreeding depression. Long-term effects are however complex, and examples spanning over multiple generations under natural conditions are scarce. The aim of this study was to test for long-term effects of natural genetic rescue in a small population of Scandinavian Arctic foxes (*Vulpes lagopus*). By combining a genetically verified pedigree covering almost 20 years with a long-term dataset on individual fitness (*n* = 837 individuals), we found no evidence for elevated fitness in immigrant F2 and F3 compared to native inbred foxes. Population inbreeding levels showed a fluctuating increasing trend and emergence of inbreeding within immigrant lineages shortly after immigration. Between 0–5 and 6–9 years post immigration, the average number of breeding adults decreased by almost 22% and the average proportion of immigrant ancestry rose from 14% to 27%. Y chromosome analysis revealed that 2 out of 3 native male lineages were lost from the gene pool, but all founders represented at the time of immigration were still contributing to the population at the end of the study period through female descendants. The results highlight the complexity of genetic rescue and suggest that beneficial effects can be brief. Continuous gene flow may be needed for small and threatened populations to recover and persist in a longer time perspective.

Inbreeding can result in lowered fitness (i.e., inbreeding depression) by increased expression of deleterious recessive alleles, or through loss of heterozygosity at loci with heterozygote advantage (Keller and Waller 2002; Hedrick and Garcia-Dorado 2016). This is a major threat to small populations and the extinction vortex model suggests that inbreeding and genetic drift could ultimately drive a population to extinction (Gilpin and Soulé 1986). To mitigate inbreeding depression, these effects need to be reduced or reversed. Deleterious recessive alleles can decrease in frequency when exposed to natural selection (i.e., purging; Charlesworth and Willis 2009) or following introduction of new genetic material from gene flow of unrelated individuals (Tallmon et al. 2004; Whiteley et al. 2014). A pre-requisite for genetic rescue to occur is that inbreeding depression is the primary driver of lowered fitness which in turn lowers population growth (e.g., Hasselgren et al. 2018). Immigration and gene flow into a population suffering from inbreeding depression can result in genetic rescue by heterosis, that is, an elevation of individual fitness in outbred individuals (Chen 2010), which promotes population growth (Tallmon et al. 2004). Previous studies have shown that genetic rescue can stem from the immigration of only a few individuals (Vila et al. 2003; Heber et al. 2013; Frankham 2016; Hasselgren et al. 2018) and facilitates the recovery of small, isolated populations suffering from inbreeding depression (Bell et al. 2019).

In times of accelerated population declines, an in-depth understanding about the impact of inbreeding depression and genetic rescue over longer time frames is crucial. Genetic rescue under natural conditions have been documented in a number of mammal populations and despite empirical support for genetic rescue effects in first-generation immigrant offspring (immigrant F1; Johnson et al. 2010; Åkesson et al. 2016; Hasselgren et al. 2018; Poirier et al. 2019), studies on its persistence in later generations are more scarce (but see Hogg et al. 2006; Johnson et al. 2010). This is problematic because long-term effects may be complex (Tallmon et al. 2004). Genetic rescue effects may be short-lived simply because heterozygosity peaks in immigrant F1 and decreases thereafter, meaning that beneficial effects will be most prominent in the first generation and level off in more distant descendants (Tallmon et al. 2004). Furthermore, immigrants can introduce detrimental recessive alleles that become expressed in the native population through inbreeding (Whiteley et al. 2014, Hedrick and Garcia-Dorado 2016, Bell et al. 2019), and these alleles are generally not expressed until second or third generation immigrant offspring (immigrant F2, F3; Tallmon et al. 2004). Also, there are records of a natural immigration event into an extremely inbred population where high success of immigrants and their descendants was followed by genetic sweep (i.e., turnover in population ancestry; Adams et al. 2011). Alternatively, benefits of outcrossing can be maintained or even greater in F2 and F3 generations than in F1 due to maternal effects on offspring fitness. This is explained by a reduction in inbreeding in mothers of F2 and subsequent generations but not in mothers of F1 generations (Frankham 2015).

The Scandinavian Arctic fox population has functioned as a model system to investigate inbreeding depression and genetic rescue effects under natural conditions (Norén et al. 2016; Godoy et al. 2018; Hasselgren et al. 2018). Intense hunting caused a major population bottleneck in the late 19th century (Lönnberg 1927) and despite legal protection, the population failed to recover due to disruptions in natural prey cycles, as well as competition and predation of northwards expanding red foxes (*Vulpes vulpes*; Hersteinsson and MacDonald 1992; Angerbjörn et al. 2013; Elmhagen et al. 2017). Moreover, fragmentation into smaller subpopulations (Dalén et al. 2006; Hemphill-Keeling et al. 2020) has increased the vulnerability to factors connected to the small population size (Loison et al. 2001). In the southernmost subpopulation in Sweden, a pedigree analysis revealed inbreeding levels corresponding to half-sib matings and inbreeding depression in fundamental life-history traits (Norén et al. 2016). However, the situation improved when 3 male Arctic foxes, released from the Norwegian captive breeding programme to a Norwegian area, unexpectedly entered the population in 2010 and 2011. The immigrants promoted a genetic rescue effect which consequently increased the number of founders from 6 to 9. In response, the inbreeding coefficient declined by 43% within 5 years and the population more than doubled in size. Immigrant F1 displayed increased first-year survival and breeding success compared to inbred native offspring (Hasselgren et al. 2018).

Whether the genetic rescue effect persisted beyond the immigrant F1-generation could however not be established (Hasselgren et al. 2018). An important factor potentially influencing the outcome of genetic rescue in this population is that 2 of the immigrants were brothers, and these had substantially higher reproductive success than the third unrelated immigrant male (Hasselgren et al. 2018). Genetic rescue may thus have been short-lived and followed by a genetic sweep and loss of both native and immigrant lineages, which again would put the population at risk of continued inbreeding and associated fitness consequences (Adams et al. 2011; Hedrick and Garcia-Dorado 2016). In this study, we test the persistence of genetic rescue effects across F1–F3 generations with specific focus on inbreeding levels and fitness. We also investigate the founder contributions and occurrence of genetic sweep following immigration using a combination of pedigree analysis based on autosomal and Y chromosome genotypes and a long-term dataset on first-year survival. Based on empirical findings from other species, we expect genetic rescue to be most pronounced in the F1 generation and thereafter decrease following high success of immigrant offspring and increased inbreeding in later generations.

## Methods

### Study System

The study population resides in Helagsfjällen (Helags), Jämtland county (62°N, 12°E; 3400 km^2^), an area located in the most southern part of the Swedish mountain tundra. The population was close to extinction in the 1990s, but since 2001 reproductions have occurred on a regular basis following return of regular prey cycles and conservation actions (Angerbjörn et al. 2013). The detailed individual monitoring of the population started in 2001 with 4 reproductive adults (2000-019-001, 2001-033-003, 2001-020-021 and Male 1; Table 1) and 2 litters (Norén et al. 2016). The population underwent a demographic increase during 2000–2009, which was accompanied by a tenfold rise in inbreeding levels corresponding to an average *f* = 0.125 (Norén et al. 2016).

**Table 1.** Representation of native founders (2000-019-001, 2001-033-003, 2001-020-021, 2004-024-044, 2005-024-001, Male 1 and Male 2) and Norwegian immigrants (N1, N2 and N3) during 2016–2019

Founder	Sex	Haplotype	2016	2017	2018	2019
2000-019-001	Male	H4	1.00	0.86	0.94	1.00
2001-033-003	Female	-	1.00	0.86	0.94	1.00
2001-020-021	Female	-	0.50	0.57	0.69	0.75
2004-024-044	Male	H1	0.00	0.57	0.56	0.33
Male1	Male	H3	0.50	0.79	0.69	0.75
Male2	Male	H1	0.50	0.79	0.88	0.75
N1	Male	H1	1.00	0.50	0.75	0.33
N2	Male	H1	0.00	0.57	0.56	0.33
N3	Male	H2	0.00	0.07	0.06	0.17
No. of litters			2	14	16	12

Values represent the proportion of litters each year that are related to each founder or immigrant when tracing both female and male lineages via pedigree data. Number of litters are litters with at least one known parental lineage that could be traced to a founder or immigrant.

The Scandinavian Arctic fox mainly preys on cyclic voles (*Myodes spp*.) and Norwegian lemmings (*Lemmus lemmus*; Angerbjörn et al. 2004). Rodent cycles generally occur in 3- to 4-year intervals (Angerbjörn et al. 2001; Henden et al. 2009; Elmhagen et al. 2011) and the Arctic fox population fluctuates heavily as a response (Angerbjörn et al. 1995; Angerbjörn et al. 2004). All known dens (*n* = 71) are visited each summer to document survival and reproductive success, and cubs are captured and ear-tagged with unique colour-coded tags with individual numerical codes. An average of 93% (±0.039) of all observed litters were sampled each year. Of all individuals, 68% were sampled during 2001–2013 (Meijer et al. 2013) and 60% were sampled during 2014–2019. Un-tagged adults were ear-tagged and sampled when captured. During ear-tagging, a skin biopsy from the ear was collected for genetic analysis.

The closest neighboring subpopulation is situated c. 40 km away, on the Norwegian side of the border. This is a small population, intensively monitored with remote cameras which records emigrants from the study population via their ear-tags. Other adjacent subpopulations are located 100–180 km away and are monitored with remote cameras, direct observations and/or DNA collection.

### Norwegian Captive Breeding Project

The Norwegian captive breeding project was established in its current form in 2005. The purpose of the project is to restore connectivity between fragmented subpopulations and counteract negative effects connected to the small population size (Landa et al. 2017). Offspring to captive parents are released to the wild along with their litter siblings at an age of 8 months (Landa et al. 2017). The breeding program includes genetic representation from most wild Scandinavian sub-populations and breeding pairs are combined with the purpose of avoiding inbreeding (Landa et al. 2017). Between 2006–2019, 425 foxes originating from the Norwegian captive breeding project were released to the wild (Ulvund et al. 2020). Releases have only been made on the Norwegian side of the border (Ulvund et al. 2020) and captive-bred foxes recorded in Swedish sub-populations represent foxes emigrating from the release site (e.g., Hasselgren et al. 2018).

### Genetic Analyses

DNA was isolated from ear-tissue collected during ear-tagging. Sample storage and DNA extraction were conducted in accordance with Norén et al. (2016). Microsatellite data were assembled from 10 polymorphic microsatellite loci from a total of 837 Arctic foxes that had been ear-tagged between 2001 and 2019. The dataset was composed of previously published genotypes of 691 individuals (2001–2009: Norén et al. 2016; 2010–2015; Hasselgren et al. 2018), and unpublished data on 146 individuals (2016–2019). Locus CPH15 (Fredholm and Winterø 1995) was replaced with locus 606 (Ostrander et al. 1995) in samples analyzed in 2010 and onwards. PCR reactions followed the procedure described in Hasselgren et al. (2018). Microsatellite alleles were determined using LIZ-500 size standard (Thermo Fisher Scientific, Waltham, MA) on an ABI3730 capillary sequencer (Applied Biosystems, Foster City, CA). Microsatellite alleles were called using the PeakScanner 1.0 software (Applied Biosystems). We tested for signatures of genotyping errors and presence of null alleles in each time period in MICROCHECKER (van Oosterhout et al. 2004) using 1000 replicates and a 95% confidence interval (CI).

### Pedigree Construction

Parents of foxes born 2016–2019 were determined by first using the exclusion principle of visually observed adults. The exclusion principle is based on the criteria that offspring inherit one allele from each parent and that a litter of full-siblings can only display 4 alleles at one given locus. The multi-locus genotypes of ear-tagged adults visually observed at a den with cubs were compared to the genotypes of the cubs (Norén et al. 2012). Second, we inferred relationships in the software COLONY v.2.0 (Jones and Wang 2010). The following parameters were set: diploidy, dioecy, occurrence of polygamy for both males and females, and occurrence of inbreeding. Foxes born further back in time than 2 full rodent cycles (i.e., 8 years or older) were excluded as candidate parents. The pedigree data were combined with an already published dataset covering foxes born 2001–2015 (Norén et al. 2016; Hasselgren et al. 2018). Individuals without known parental origin that generate new genetic lineages in the pedigree are referred to as “founders” (i.e., pedigree founders following Garbe and Da 2008, Table 1). Individual inbreeding coefficients (*f*) were derived for litters with at least 2 full generations of known parentage (Balloux et al. 2004) using the software Pedigraph v.2.2 (Garbe and Da 2008). Progeny of a native and an immigrant (native × immigrant) were assigned as immigrant F1, progeny of a native and an immigrant F1 (native × F1) were assigned as F2 and those of a native and an immigrant F2 (native × F2) were assigned as F3. Individuals were assigned as native if no immigrant ancestry could be traced in their origin.

### Fitness Measure

We used first-year survival as a proxy for fitness (Norén et al. 2016; Hasselgren et al. 2018). Survival was determined as whether an individual survived its first year or not (i.e., 1 or 0) and was assembled from genetic parentage assignments, visual observations, and remote camera photos. Re-sightings of ear-tagged cubs at dens is relatively high. Average juvenile summer survival (measured over 40 days) is 96% during high small rodent density and 51% during low small rodent density (Erlandsson et al. 2017). Juvenile first-year survival estimates (mainly based on visual re-observations of ear-tagged foxes) varies between approximately 20% during low rodent density to 40% during high small rodent density (Hasselgren et al. 2018), but may be as low as 10% (Meijer et al. 2008). In this dataset, 43% of first-year survivors were based on visual observations, 27% were based on remote camera photos and 30% were based solely on genetic assignment. Visual observations and remote camera photos of first-year survivors were also verified through genetic assignment when litters were present. A fox that had not been observed for a full rodent cycle and had not appeared in any of the other subpopulations was assumed to be dead. Since Arctic fox survival is strongly affected by the rodent cycle (Meijer et al. 2008, 2013), the phase of the rodent cycle each fox was born in was considered in the statistical analyses. To maintain statistical power, rodent phases were merged together into “increase” or “decrease” phases, which was determined by whether each litter was born in a year of increasing or decreasing food abundance (Hasselgren et al. 2018). Rodent densities were inferred from standardized snap trapping carried out in the field during summer inventories (Angerbjörn et al. 2013; Erlandsson et al. 2017).

### Statistical Analyses

Population size was estimated as the minimum number of breeding adults (Angerbjörn et al. 2013). Probability of identity (Waits et al. 2001) was calculated for the whole population (*n* = 837) in GenAlEx v.6.5 (Peakall and Smouse 2012). A standardized measurement of multi-locus heterozygosity (sMLH) was calculated for each fox according to Coltman et al. (1999). Three time periods were used to investigate the effects of immigration on autosomal genetic variation in the population: 2001–2009 (pre-immigration), 2010–2015 (post-immigration, short term), and 2016–2019 (post-immigration, long term). The last F1 litter was born in 2015 and during 2016–2019, mainly F2 and F3 litters were born, which is the underlying reason for separating the time periods in the analyses. In addition, 6 F4 and 1 F5 litters were born during 2018–2019, but these were not included in the analyses. GenAlEx v.6.5 was used to test for deviations from Hardy-Weinberg proportions between the 3 periods. Allelic richness (Hughes et al. 2008) was calculated in FSTAT v.2.9.3.2 (Goudet 2002). Differences in allelic richness and sMLH between periods were investigated by conducting a non-parametric Kruskal–Wallis tests in R v.1.2.5033. A Kruskal–Wallis test was also used to investigate potential differences in sMLH between native offspring, immigrant F1, and immigrant F2+F3 born 2010–2019. Dunn’s test was used for post-hoc comparisons. An allele frequency plot for the 3 time periods was manually constructed where alleles with 5% prevalence or less was classified as rare alleles. To visualize the changes in ancestry over time, a Bayesian MCMC approach was implemented in Structure 2.3.4 (Pritchard et al. 2000). To define the most likely number of genetic clusters in the full data set, we used 100 000 burn-in steps and 1 million iterations without any prior population information. K ranged from 1 to 10 clusters and each setting was replicated 3 times. The most likely number of clusters were identified using the Evanno ΔK approach (Evanno et al. 2005). After defining the number of genetic clusters, each individual was a priori assigned to its specific time period and the number of clusters was set to *K* = 3. We used 100 000 burn-in steps and 1 million iterations using the prior population information approach.

We used a generalized linear mixed-effect model to investigate whether first-year survival differed between native offspring, immigrant F1, and immigrant F2+F3. Sex as well as which rodent phase a fox was born in were set as fixed effects. Natal den site was set as a random effect to control for territorial quality. First-year survival was analyzed for foxes born 2010–2015. Four years (i.e., one full rodent cycle) of monitoring was needed to properly evaluate the foxes’ survival. Individuals born after 2015 were thus excluded from the analysis due to a lack of fitness data available at the time.

### Y Chromosome Genotyping, Founder Analysis and Genetic Sweep

To screen for variation in the male lineage, one male from each litter born between 2001–2019 (*n* = 144) was genotyped in 3 Y-chromosome-linked microsatellite loci: VVY15, VVY16, VVY17. Primers were originally developed for the red fox (Rando et al. 2017). Extra-pair paternity in the Scandinavian Arctic fox is, in comparison to Arctic fox populations in stable resource-rich areas, low and would likely be detected through autosomal genotyping (Norén et al. 2012). Additionally, 24 males originating from Svalbard, Canada and Iceland, as well as from a northern subpopulation in Sweden (Vindelfjällen/Arjeplog) were included as reference material.

Y chromosome alleles were manually assembled into haplotypes and visualized through a median-joining network constructed in NETWORK 5.0 (www.fluxus-engineering.com; Bandelt et al. 1999). The contribution of different genetic lineages over time was investigated by combining unique Y chromosome haplotypes with autosomal pedigree data to also account for female descendants. Founder analysis with the use of pedigree data was done in PedScope (www.pedscope.co.uk; Tenset Technologies Ltd.).

Immigrant ancestry was calculated for litters included in the pedigree as the expected proportion of genes from a given individual carried by its descendants (Lacy 1989). Based on the proportion of immigrant ancestry in each litter, we subsequently estimated the proportion of immigrant ancestry for every year. Finally, we calculated the proportion of litters related to the immigrants for each year, and for this analysis, we also included litters with only one grandparent known.

## Results

### Pedigree and Genetic Variation

Of the 146 Arctic foxes that were ear-tagged in 2016–2019, 144 were successfully genotyped in 10 loci. Inbreeding coefficients (*f*) could be derived for 25 out of 48 sampled litters born during the same time period. A total of 213 litters were recorded since monitoring began in 2001, of which 700 individuals from 140 litters (66%) were included in the final pedigree (2001–2019). A majority of these litters (78%) could be traced to at least one founder. The probability of identity was *P*_ID_= 7 × 10^–4^ assuming a full-sibling relationship and *P*_ID_ = 5.8 × 10^–8^ assuming they were unrelated.

**Figure 1.**
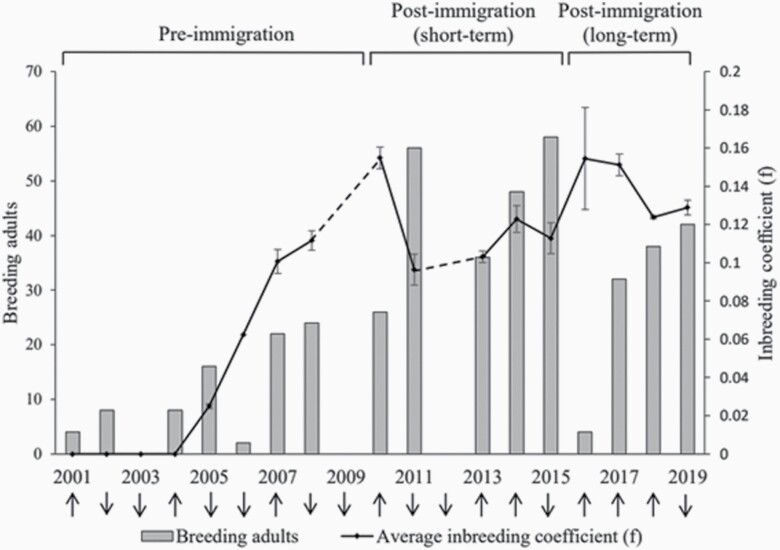
Average inbreeding coefficient (*f*) with variance for Arctic fox litters (line) and the number of breeding adults (bars) in 2001–2019. Dashed lines represent years with no data due to no reproduction. Arrows represent increasing or decreasing small rodent densities at cub birth.

In 2010, when the first gene flow event occurred, the average inbreeding coefficient (*f*) of cubs peaked at *f* = 0.155 (Figure 1). In 2011, inbreeding decreased by 38% to *f* = 0.096, but increased again in the following years. In 2016, inbreeding levels reached *f* = 0.154. However, only 2 litters were born this year, one of which was the result of a parent-offspring mating within an immigrant lineage. The average inbreeding coefficient subsequently declined to *f* = 0.124 in 2018 and *f* = 0.129 in 2019. A total of 12 backcrosses within immigrant lineages were recorded, of which the first one occurred in 2014. Among these, 2 cases were parent-offspring matings (N1-F1 × N1-F2 and N1-F1 × N1-F2) and other 2 cases were matings between half-siblings (N3-F1 × N3-F1) and full-siblings (and N2-F1 × N2-F1), where N1-N3 represent the 3 immigrants. The remaining 8 cases were between more distantly related individuals. An average of 29 adults reproduced in 2016–2019, which equals a 22% decline from the average of 37 adults that reproduced in 2010–2015.

Allelic richness increased significantly from 3.78 (±0.39) in 2001–2009 to 4.90 (±0.74) in 2010–2015 (χ ^2^ = 2.707; *P* = 0.003) and remained significantly higher at 5.38 (±0.91) in 2016–2019 (χ ^2^ = 2.482; *P* = 0.007). There was no significant change in allelic richness between the 2 post-immigration periods (χ ^2^ = 0.226; *P* = 0.411). Further, heterozygosity (sMLH) was significantly higher in immigrant F1 (µ = 1.058 ± 0.072; *n* = 53) compared to both native offspring (µ = 0.947 ± 0.041; *n* = 152; χ ^2^ = 2.407; *P* = 0.008) and immigrant F2+F3 (µ = 0.955 ± 0.030; *n* = 268; chi2 = 2.262; *P* = 0.012). There was no significant difference in sMLH between native offspring and immigrant F2+F3 (χ ^2^ = 0.264; *P* = 0.396) or between any of the 3 time periods (*n* = 829; χ ^2^ = 1.649; *P* = 0.438).

Prior to immigration (2001–2009), 5 loci displayed significant heterozygote excess (Supplementary Table S1). After immigration, 4 loci displayed heterozygote deficiency in 2010–2015, and 5 loci deviated in 2016–2019. Of these, one locus showed heterozygote excess and the remaining 4 heterozygote deficiency. No deviating loci showed a consistent pattern across the different time periods. No systematic occurrence of null alleles or genotyping errors was detected across time periods (95% CI). However, an allele frequency spectrum plot showed that the number of rare alleles (<5%) increased shortly after immigration (from 10 rare alleles during 2001–2009 to 22 rare alleles during 2010–2015) but thereafter slightly decreased again (17 rare alleles during 2016–2019; Supplementary Figure S1).

**Figure 2.**
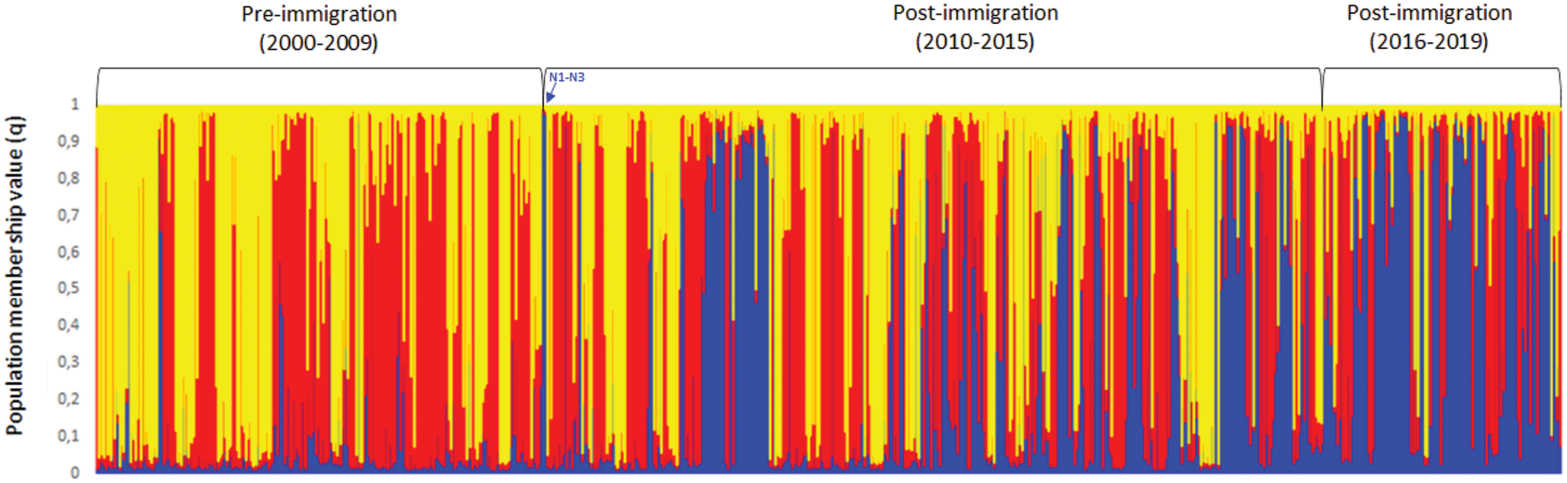
Structure bar plot showing the population membership values (*q*) for each individual and time period for *K* = 3 with prior population information. Red, yellow, and blue represent different genetic clusters. N1–N3 represent the 3 immigrants with genetic profiles assigned to the blue genetic cluster.

Based on STRUCTURE simulations without prior population information for *K* = 2–10, the Evanno approach demonstrated that *K* = 3 was the most likely number of clusters over 3 replicates (Supplementary Figure S2a). A gradual change in genetic composition following immigration in 2010 was shown (Supplementary Figure S2b). The Structure approach using *K* = 3 with prior population information of sampling period for each individual, also showed a change in genetic composition over time (Figure 2). This is illustrated from a shift from the red and yellow cluster represented in the native population prior to gene flow, to an increase of the blue cluster related to the genetic profiles of the immigrants N1–N3 (Figure 2).

### First-Year Survival

**Figure 3.**
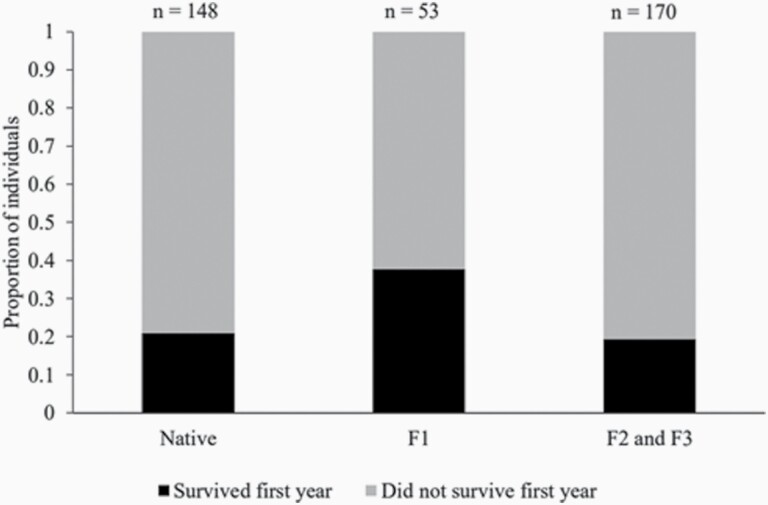
First-year survival of native offspring (no immigrant ancestry; *n* = 148), first generation immigrant offspring (F1; *n* = 53), and second- and third-generation immigrant offspring (F2 and F3; *n* = 170) in Arctic foxes born 2010–2015.

The analysis included 145 native offspring, 53 immigrant F1 and 166 immigrant F2+F3 born in 2010–2015 (Supplementary Table S6). First-year survival was twice as high in immigrant F1 compared to native offspring (*Z* = 3.071; *P* = 0.003; Figure 3) and F2+F3 (*Z* = 3.165; *P* = 0.002). There was however no difference in survival between native offspring and immigrant F2+F3 (*P* = 0.996). Irrespective of their ancestry, 26% of cubs survived their first year during increasing rodent densities compared to 19.6% during decreasing rodent densities (*Z* = −2.026; *P* = 0.044). Further, there was a trend for lower survival for males compared to females (*Z* = −1.919; *P* = 0.055). Male first-year survival was 18.4% and female survival was 26.7%.

### Founder Representation and Genetic Sweep

Y chromosome genotypes could be determined for 133 out of 144 males from the study population. Loci VVY15 and VVY17 were polymorphic before immigration occurred (Supplementary Table S2). Gene flow initially increased variation in VVY17 owing to one allele introduced by the unrelated immigrant in 2010. Five years after immigration, however, VVY15 was no longer polymorphic and variation in VVY17 had decreased by one-third through loss of one native allele (Supplementary Table S2). The introduced allele became increasingly more common over time.

**Figure 4.**
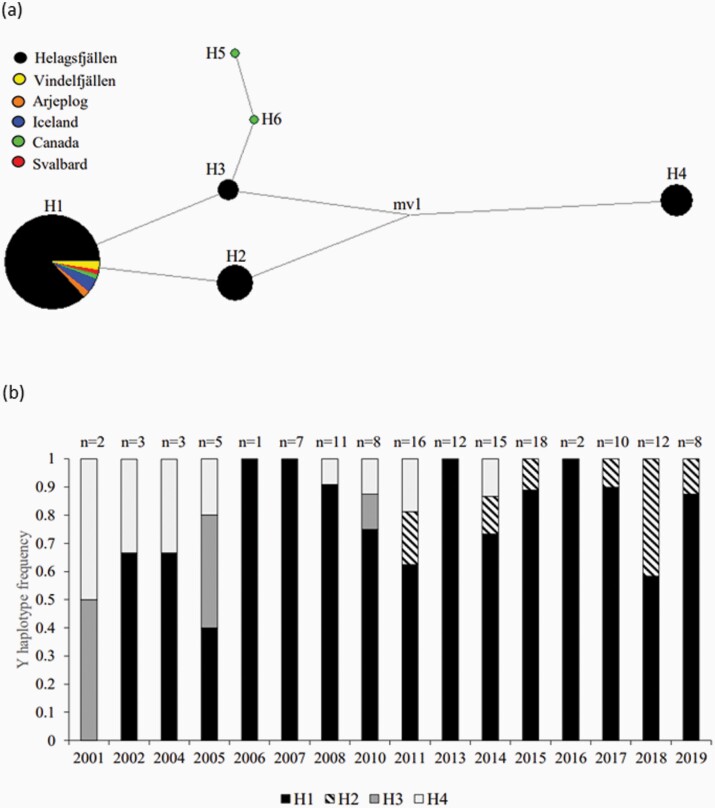
(**a**) Median-joining network displaying the relationship between 6 Y chromosome haplotypes observed in Arctic foxes from Helagsfjällen (study population) and reference populations. Haplotype size is proportional to frequencies. Mv1 represents a median vector. (**b**) Haplotype representation in 133 males born in 2001–2019. Years without reproduction were excluded from the graph.

Four out of six constructed haplotypes were recorded in our study population and the remaining 2 were observed in Canadian samples (Supplementary Table S3). H1 was the most common haplotype in the study population across the whole study period (Figure 4a). Two out of four native male founders had this haplotype, whereas the other 2 were assigned the less frequent haplotypes H3 and H4. The 2 immigrant brothers had the already common haplotype H1 and the unrelated immigrant male introduced haplotype H2 to the population. Following gene flow, 2 out of 3 native haplotypes were lost from the population (Figure 4b).

When combining Y chromosome genotypes with pedigree data it was however established that all male founders were still genetically represented in the population gene pool in 2019 (Table 1). Furthermore, a male founder (2004-024-44), previously classified as genetically lost in 2008 (Norén et al. 2016), reappeared through a descendant in 2010. One of the 3 native female founders was lost; however, which occurred before immigration took place. Thus, as of 2019, the study population gene pool was based on a total of 9 individuals: 6 males and 3 females (Supplementary Table S4). The third female was the mother of the immigrant brothers.

**Figure 5.**
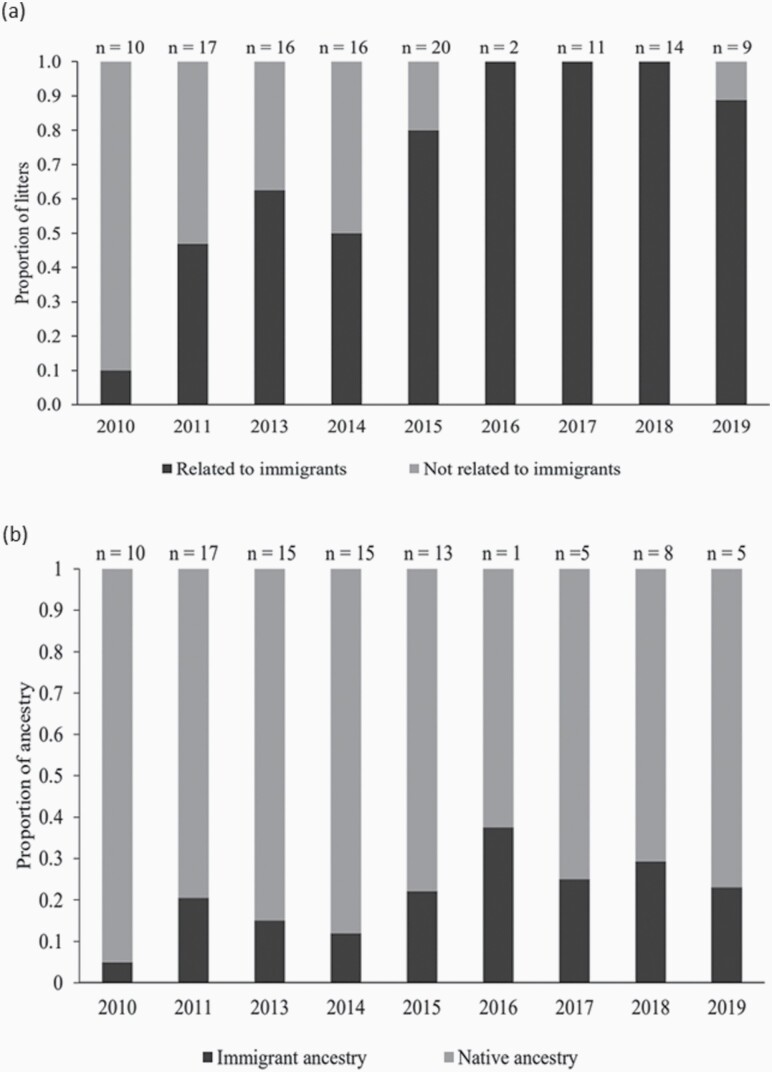
(**a**) Proportion of litters with immigrant ancestry. (**b**) Proportion of immigrant and native ancestry in litters. Estimates of immigrant ancestry was based on Lacy (1989).

Based on the updated pedigree, it was established that all except one litter with known parents born in 2016–2019 were descendants of the immigrants (Figure 5a; Supplementary Table S4; Supplementary Table S5). Genetic contribution from immigrants in 2010–2015 ranged from 0.05 to 0.22 with an average of 0.14 (Figure 5b). In 2016–2019, immigrant ancestry ranged from 0.231 to 0.375 with an average of 0.27 (Supplementary Table S4).

## Discussion

Genetic rescue has the potential to facilitate recovery of small and isolated populations suffering from inbreeding depression (Bell et al. 2019). Our knowledge on the persistence of these effects under natural conditions is however limited. The aim of our study was to investigate the persistence of genetic rescue across generations in a Scandinavian Arctic fox population (Hasselgren et al. 2018). The study population experienced steadily increasing inbreeding levels due to 9 years of isolation (Norén et al. 2016), followed by gene flow that resulted in decreasing inbreeding levels (Hasselgren et al. 2018). It was assumed that the decrease continued for several years (Hasselgren et al. 2018), but the updated pedigree revealed another pattern. The yearly inbreeding coefficient began to fluctuate shortly after immigration. In 2016 and 2017, inbreeding levels increased to pre-immigration levels but thereafter declined, and by the end of the study period, corresponded to a level of half-sibling mating. Although spanning over a longer time frame, a similar fluctuating pattern after gene flow was observed in the wolf population on Isle Royale, North America prior to its collapse (Adams et al. 2011; Hedrick et al. 2014, 2019). Apart from the fluctuations, our results show an overall increase in inbreeding levels, mainly explained by the close inbreeding that occurred within immigrant lineages already 4 years after immigration. The observed occurrences of inbreeding included matings between parent-offspring and natal siblings in 2016 and 2017. Even though inbreeding estimates are based on a limited number of microsatellite loci which is a general caveat for pedigree construction (Kardos et al. 2015), we consider the data robust. Hasselgren et al. (2021) found a strong correlation between pedigree inbreeding coefficients and genomic-based inbreeding estimates in the study population.

An important question was whether the positive fitness effect in immigrant offspring remained across generations. Evidence for genetic rescue in immigrant F1 following gene flow was documented by Hasselgren et al. (2018) and in agreement, we found immigrant F1 to have 2× higher survival compared to inbred native foxes. However, there was no difference in first-year survival between immigrant F2+F3 and native inbred offspring, which implies that the genetic rescue effect was short-lived. This is further supported immigrant F1 being more heterozygous compared to both native and immigrant F2+F3 offspring. Frankham (2016) suggested that genetic rescue may persist until the F3-generation, but this was not the case in our study. Instead, results from this study are concordant with data on a natural population of bighorn sheep residing in Ram Mountain, Alberta, United States (Poirier et al. 2019). However, our analysis was limited to first-year survival, which means that fitness benefits may still be apparent in other parts of the life cycle.

Initial positive fitness effects following gene flow is thought to be due to heterosis of immigrant offspring, which occurs when immigrant alleles mask deleterious recessive alleles present in the native population, or when heterozygosity is brought back to loci with heterozygous advantage (Tallmon 2004). Heterozygosity is expected to be highest in immigrant F1 and decline thereafter (Tallmon 2004). In absence of maternal effects of inbreeding (Frankham 2015), genetic rescue is predicted to be most profound in immigrant F1 and level off in more distant descendants. It is however also possible that gene flow into an extremely small population can introduce recessive deleterious alleles that become expressed in immigrant F2 and F3 (Whiteley et al. 2014; Hedrick and Garcia-Dorado 2016; Bell et al. 2019; Kyriazis et al. 2021, but see Ralls et al. 2020). Such consequences are however only expected when inbreeding levels in the recipient population are already high, population size low and gene flow occurs as one single event (Ralls et al. 2020).

Shortly after gene flow, we recorded inbreeding events between the immigrant lineages, which suggests that continued inbreeding is a likely future scenario, especially if gene flow is not continuous (e.g., Ralls et al. 2020). The consequences of inbreeding are however be challenging to predict, but through genome sequencing, the genetic basis of inbreeding depression as well as the risk of genetic sweep and/or introduction of deleterious alleles following gene flow can be addressed (Supple and Shapiro 2018). Furthermore, including a perspective of adaptive variation into captive breeding program design facilitates a way to select genetically appropriate individuals prior to release and increase the chances for successful genetic rescue (Supple and Shapiro 2018). Furthermore, the fact that 2 of the immigrants were full-siblings is likely influencing the outcome and for planned genetic rescue events, a thorough selection of released individuals that avoid releases of close relatives is recommended (Frankham et al. 2017). Another option, apart from captive releases, would be to focus conservation actions to restore connectivity between fragmented subpopulations and increase chances of natural gene flow between historically connected populations (Hemphill-Keeling et al. 2020).

A cornerstone of genetic rescue is increased population growth (Tallmon et al. 2004; Bell et al. 2019). Genetic rescue of the study population can be considered short-term also in this aspect. The number of breeding adults doubled shortly after immigration (Hasselgren et al. 2018) and an average of 37 adults reproduced during 2010–2015. In the following period (2016–2019), an average of 29 adults reproduced, which equals a 22% decline. Disentangling the causes responsible for the population decline is however difficult as population development is influenced by both environmental and genetic factors, as well as conservation actions (Angerbjörn et al. 2013). Furthermore, since the study population has a cyclic demography, the outcome of immigration and strength of a potential genetic rescue effect is likely phase-dependent (Norén and Angerbjörn 2014). Arctic fox juvenile survival is influenced by prey abundance (Tannerfeldt et al. 1994), which means that the immigrant success is expected to be highest if they arrive to the recipient population during an increase phase. This was the case for the Norwegian immigrants and the timing of their arrival was likely contributing to the initially high success (Hasselgren et al. 2018).

Previous studies have demonstrated that genetic sweep following gene flow may under specific circumstances have a profound impact on both individual fitness and population demography (Adams et al. 2011; Robinson et al. 2019, but see Ralls et al. 2020). We found that 2 out of 4 native male haplotypes were lost following immigration. Low frequency of H3 and H4 implied an already imminent risk of these haplotypes being lost by genetic drift (Nei et al. 1975). However, high survival and high breeding success early in life of immigrant F1 resulted in high immigrant success (Hasselgren et al. 2018). All except one litter born in 2016–2019 were immigrant descendants, meaning that even though the 2 native haplotypes may have eventually been lost due to genetic drift or demographic stochasticity, the success of the immigrants likely accelerated the process. The immigrant brothers were assigned haplotype H1, which, in combination with the already high frequency of this haplotype before immigration, could explain its persistence (Nei et al. 1975). Haplotype H2 was introduced in 2011 by the unrelated male. Pedigree data had previously suggested that this male was only related to 5% of the litters born in 2015 (Hasselgren et al. 2018), but his success appears to have been underestimated using only autosomal data.

Our analysis of founder contribution demonstrated that all male founders were still genetically represented in 2019. The pedigree analysis revealed that one of the native male founders who was previously thought to have been genetically lost in 2008 (Norén et al. 2016) re-appeared through a descendant in 2010 and was still contributing to the gene pool in 2019. Also, the ancestry of the 2 males whose Y chromosome lineages had been lost, was passed on through female descendants. On the female side, 2 out of 3 native founders were also still genetically represented in 2019. The only genetic lineage that was lost since the detailed individual monitoring was initiated in 2001 was a female who produced one litter in 2005 of which no offspring reproduced (Norén et al. 2016). Conclusively, at the end of the study period, the population gene pool was based on by 6 males and 3 females.

We demonstrate that the genetic contribution from immigrants almost doubled over time, from an average of 14% in 2010–2015 to 27% in 2016–2019. Related to this, we recorded a gradual change in individual genetic composition after the immigration events in 2010, which likely is a consequence of high genetic impact of immigrants (Figure 2). Genetic rescue is associated with a potential cost of reduced native ancestry, which can lower genetic variation and swamp local adaptations (Frankham 2015). Ralls et al. (2018) however argue this to be a minor issue since small populations will often not be well-adapted to their environment due to genetic drift and anthropogenic changes in local conditions. A turnover in ancestry may however increase the risk of inbreeding to continue, which was the case in Isle Royale wolf population. Immigrant ancestry constituted 56% only 2.5 generations after the gene flow event (Adams et al. 2011). Following initial genetic rescue, the extremely high immigrant success may have contributed to the population to eventually collapsing (Robinson et al. 2019). In contrast, however, examples from Florida panthers (Johnson et al. 2010) and bighorn sheep in Montana (Hogg et al. 2006) show that it is possible to have moderate or high turnover in ancestry parallel with positive demographic effects. In the light of the 3 examples mentioned above, however, genetic sweep in our study population can be considered weak. Especially considering that all native founders present at the time of immigration were still contributing to the population gene pool at the end of the study period. Nevertheless, immigrant ancestry will gradually increase if inbreeding between immigrant descendants continue.

## Conclusions

This study highlights the complex nature of genetic rescue and shows that even though initial effects on fitness and demography are strong, they may not persist across generations. This supports the background theory of genetic rescue being more pronounced in the F1 generation and adds to empirical evidence from other species. Inbreeding levels can rapidly increase in absence of continuous gene flow and from initial high success of immigrant lineages. The risk of inbreeding depression may thus return through increased homozygosity of deleterious alleles. High success of immigrants and their descendants can come at the cost of reduced native genetic diversity, but we have shown that native founders are not necessarily lost in this process. Together, our results suggest that a singular pulse of gene flow is not enough to mitigate inbreeding depression in a longer time perspective. Absence of additional gene flow into the population will likely result in a downward spiral of further intensified inbreeding depression over time.

## Supplementary Material

esab011_suppl_Supplementary_Material

## Data Availability

Data is publicly available in Dryad, doi:10.5061/dryad.hx3ffbgdk
