# Supplementary material for: Low Persistence of Genetic Rescue Across Generations in the Arctic Fox (Vulpes lagopus)
Source: J Hered. 2021 Mar 19;112(3):276–85. doi: 10.1093/jhered/esab011 (PMC8141685; doi:10.1093/jhered/esab011)
Supplement: esab011_suppl_Supplementary_Material [file esab011_suppl_supplementary_material.docx]

**Supplementary file.**

**Table S1** Expected (He) and observed (Ho) heterozygosity in ten loci in Arctic foxes born 2001-2009 (n = 253), 2010-2015 (n = 434), and 2016-2019 (n = 144). P-values in bold show a significant deviation from HWE. Foxes born in the first period were not genotyped in locus 606 (Norén et al. 2016).

|  | Pre-immigration | | | |  |  |  |  |  | Post-immigration | | | |  |  |  |
| --- | --- | --- | --- | --- | --- | --- | --- | --- | --- | --- | --- | --- | --- | --- | --- | --- |
|  |  | 2001- 2009 | |  |  |  |  | 2010–2015 | |  |  |  | 2016–2019 | | |  |
| Locus | No. of alleles | He | Ho | p-value | | No. of alleles | He | | Ho | p-value | No. of alleles | He | Ho | p-value | | |
| CXX173 | 4 | 0.62 | 0.68 | 0.256 | | 4 | 0.58 | | 0.58 | 0.593 | 4 | 0.48 | 0.44 | 0.204 | | |
| CPH3 | 5 | 0.73 | 0.77 | **0.021** | | 8 | 0.73 | | 0.72 | **0.000** | 8 | 0.80 | 0.82 | 0.428 | | |
| 606 | - | - | - | - | | 6 | 0.54 | | 0.61 | 0.052 | 6 | 0.52 | 0.46 | **0.000** | | |
| 671 | 4 | 0.73 | 0.84 | **0.001** | | 5 | 0.69 | | 0.70 | 0.625 | 4 | 0.66 | 0.71 | 0.470 | | |
| 758 | 4 | 0.67 | 0.68 | 0.838 | | 5 | 0.69 | | 0.63 | **0.047** | 5 | 0.73 | 0.86 | **0.024** | | |
| 771 | 4 | 0.66 | 0.74 | **0.009** | | 6 | 0.66 | | 0.66 | 0.087 | 7 | 0.71 | 0.67 | 0.231 | | |
| CXX250 | 4 | 0.56 | 0.63 | 0.080 | | 7 | 0.65 | | 0.70 | 0.701 | 7 | 0.75 | 0.67 | **0.002** | | |
| CXX140 | 4 | 0.37 | 0.37 | 0.998 | | 6 | 0.46 | | 0.49 | 0.052 | 7 | 0.58 | 0.60 | 0.994 | | |
| CPH9 | 3 | 0.65 | 0.69 | **0.033** | | 4 | 0.64 | | 0.63 | **0.001** | 4 | 0.69 | 0.67 | **0.039** | | |
| 377 | 5 | 0.44 | 0.47 | **0.000** | | 4 | 0.57 | | 0.53 | **0.000** | 4 | 0.51 | 0.46 | **0.001** | | |
| Average |  | 0.60 | 0.65 |  | |  | 0.62 | | 0.63 |  |  | 0.64 | 0.64 |  | | |


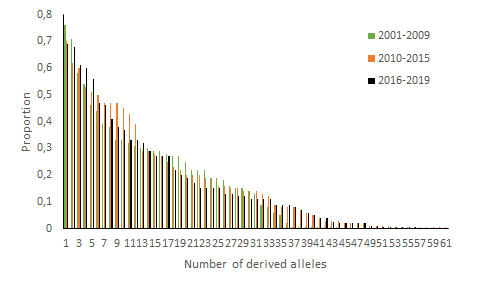


**Figure S1.** Allele frequency spectrum of overlapping loci in Arctic foxes born during 2001-2009, 2010-2015 and 2016-2019 respectively.

**Table S2** Average allele frequencies in VVY15 and VVY17 in 2001-2009 (n = 32), 2010-2015 (n = 69), and 2016-2019 (n = 32). The immigrant brothers had allele 149 and 247, whereas the third immigrant male had allele 149 and 251. Numbers in bold show allele loss/fixation.

|  |  | Pre-immigration |  | Post-immigration | |
| --- | --- | --- | --- | --- | --- |
| Locus | Allele | 2001–2009 | 2010–2015 | | 2016–2019 |
| VVY15 | 149 | 0.750 | 0.899 | | **1.000** |
|  | 153 | 0.250 | 0.101 | | **0.000** |
| VVY17 | 247 | 0.844 | 0.812 | | 0.781 |
|  | 251 | 0.000 | 0.101 | | 0.219 |
|  | 257 | 0.156 | 0.087 | | **0.000** |

**Table S3** Variation in three Y chromosome linked loci for the Helags population as well as 18 males from reference populations (n_Vindelfjällen_ = 3, n_Arjeplog_ = 3, n_Svalbard_ = 2, n_Iceland_ = 6, n_Canada_ = 4). Constructed haplotypes, named H1-H6, based on the variation in three Y chromosome linked loci where haplotype H1-H4 were observed in the Helags population in 2001-2019 (n = 138) and haplotype H5 and H6 were observed in two Canadian reference samples.

| Locus | H1 | H2 | H3 | H4 | H5 | H6 |
| --- | --- | --- | --- | --- | --- | --- |
| VVY15  alleles | 149 | 149 | 153 | 153 | 155 | 153 |
| VVY17  alleles | 247 | 251 | 247 | 257 | 247 | 247 |
| VVY16  alleles | 211 | 211 | 211 | 211 | 213 | 213 |

**Table S4** Representation of native founders (2000-019-001, 2001-033-003, 2001-020-021, 2004-024-044, 2005-024-001, Male 1 and Male 2) and Norwegian immigrants (N1, N2 and N3) during 2001-2019. The values represent the proportion of litters each year that are related to each founder. Number of litters are litters with at least one known parental lineage that could be traced to a founder or immigrant.

| Year | No. of litters | 2000-019-001 | 2001-033-003 | 2001-020-021 | 2004-024-044 | 2005-024-001 | Male1 | Male2 | N1 | N2 | N3 |
| --- | --- | --- | --- | --- | --- | --- | --- | --- | --- | --- | --- |
| 2001 | 2 | 0.50 | 0.50 | 0.50 | - | - | 0.50 | - | - | - | - |
| 2002 | 7 | 0.57 | 0.57 | 0.57 | - | - | 0.29 | 0.29 | - | - | - |
| 2003 | 1 | 1.00 | 1.00 | 0.00 | - | - | 0.00 | 1.00 | - | - | - |
| 2004 | 4 | 0.75 | 0.75 | 0.50 | 0.25 | - | 0.50 | 0.25 | - | - | - |
| 2005 | 7 | 1.00 | 1.00 | 0.71 | 0.00 | 0.14 | 0.43 | 0.43 | - | - | - |
| 2006 | 1 | 1.00 | 1.00 | 0.00 | 0.00 | 0.00 | 0.00 | 1.00 | - | - | - |
| 2007 | 10 | 1.00 | 1.00 | 0.30 | 0.10 | 0.00 | 0.30 | 0.90 | - | - | - |
| 2008 | 14 | 0.93 | 0.93 | 0.50 | 0.00 | 0.00 | 0.50 | 0.79 | - | - | - |
| 2009 | 0 |  |  |  |  |  |  |  |  |  |  |
| 2010 | 10 | 1.00 | 1.00 | 0.60 | 0.10 | 0.00 | 0.60 | 1.00 | 0.00 | 0.10 | 0.00 |
| 2011 | 18 | 0.89 | 0.89 | 0.28 | 0.28 | 0.00 | 0.28 | 0.78 | 0.06 | 0.22 | 0.22 |
| 2012 | 0 |  |  |  |  |  |  |  |  |  |  |
| 2013 | 15 | 1.00 | 1.00 | 0.53 | 0.20 | 0.00 | 0.53 | 1.00 | 0.47 | 0.13 | 0.00 |
| 2014 | 16 | 1.00 | 1.00 | 0.63 | 0.25 | 0.00 | 0.63 | 0.81 | 0.38 | 0.25 | 0.06 |
| 2015 | 21 | 0.90 | 0.95 | 0.57 | 0.33 | 0.00 | 0.57 | 0.81 | 0.33 | 0.33 | 0.14 |
| 2016 | 2 | 1.00 | 1.00 | 0.50 | 0.00 | 0.00 | 0.50 | 0.50 | 1.00 | 0.00 | 0.00 |
| 2017 | 14 | 0.86 | 0.86 | 0.57 | 0.57 | 0.00 | 0.79 | 0.79 | 0.50 | 0.57 | 0.07 |
| 2018 | 16 | 0.94 | 0.94 | 0.69 | 0.56 | 0.00 | 0.69 | 0.88 | 0.75 | 0.56 | 0.06 |
| 2019 | 12 | 1.00 | 1.00 | 0.75 | 0.33 | 0.00 | 0.75 | 0.75 | 0.33 | 0.33 | 0.17 |

**Table S5.** Number of Arctic foxes with native and immigrant ancestry sampled and included in the pedigree during 2010-2019.

|  | **2010** | **2011** | **2012** | **2013** | **2014** | **2015** | **2016** | **2017** | **2018** | **2019** |
| --- | --- | --- | --- | --- | --- | --- | --- | --- | --- | --- |
| **Native** | 41 | 34 | 0 | 18 | 25 | 28 | 0 | 0 | 0 | 2 |
| **F1** | 11 | 38 | 0 | 0 | 0 | 3 | 0 | 0 | 0 | 0 |
| **F2** | 0 | 17 | 0 | 34 | 40 | 50 | 5 | 18 | 14 | 7 |
| **F3** | 0 | 0 | 0 | 0 | 5 | 22 | 0 | 24 | 26 | 7 |
| **F4** | 0 | 0 | 0 | 0 | 0 | 0 | 0 | 0 | 8 | 4 |
| **F5** | 0 | 0 | 0 | 0 | 0 | 0 | 0 | 0 | 2 | 0 |
| **Missing** | 6 | 17 | 0 | 3 | 11 | 19 | 0 | 8 | 12 | 6 |
| **Phase** | high | low | low | high | high | low | high | high | high | low |

**Table S6.** Results from GLMM model Survival ~ Ancestry + Phase + Sex + (1 | Den), Data = Survival) where bold values indicate significant results. Asterisks show level of significance as P<0.001 ***, P<0.01 ** and P<0.05 *.

| **Fixed effects** | | | | | |
| --- | --- | --- | --- | --- | --- |
|  | **Estimate** | **Standard error** | **df** | **t-value** | **P-value** |
| **(Intercept)** | 0.49881 | 0.07348 | 58.622 | 6.788 | **6.34E-094***** |
| **Ancestry: F2+F3** | -0.2191 | 0.06921 | 71.6831 | -3.165 | **0.00227**** |
| **Ancestry: native** | -0.219 | 0.07132 | 76.4927 | -3.071 | **0.00295**** |
| **Phase: Low** | -0.0916 | 0.04522 | 293.452 | -2.026 | **0.04365*** |
| **Sex: M** | -0.0838 | 0.04365 | 358.995 | -1.919 | 0.05584 |
| **Random effects** | | | | | |
| **Groups** | **Name** | **Variance** | **Standard deviation** |  |  |
| **Den** | (Intercept) | 0.00178 | 0.04213 |  |  |
| **Residual** |  | 0.16914 | 0.41127 |  |  |


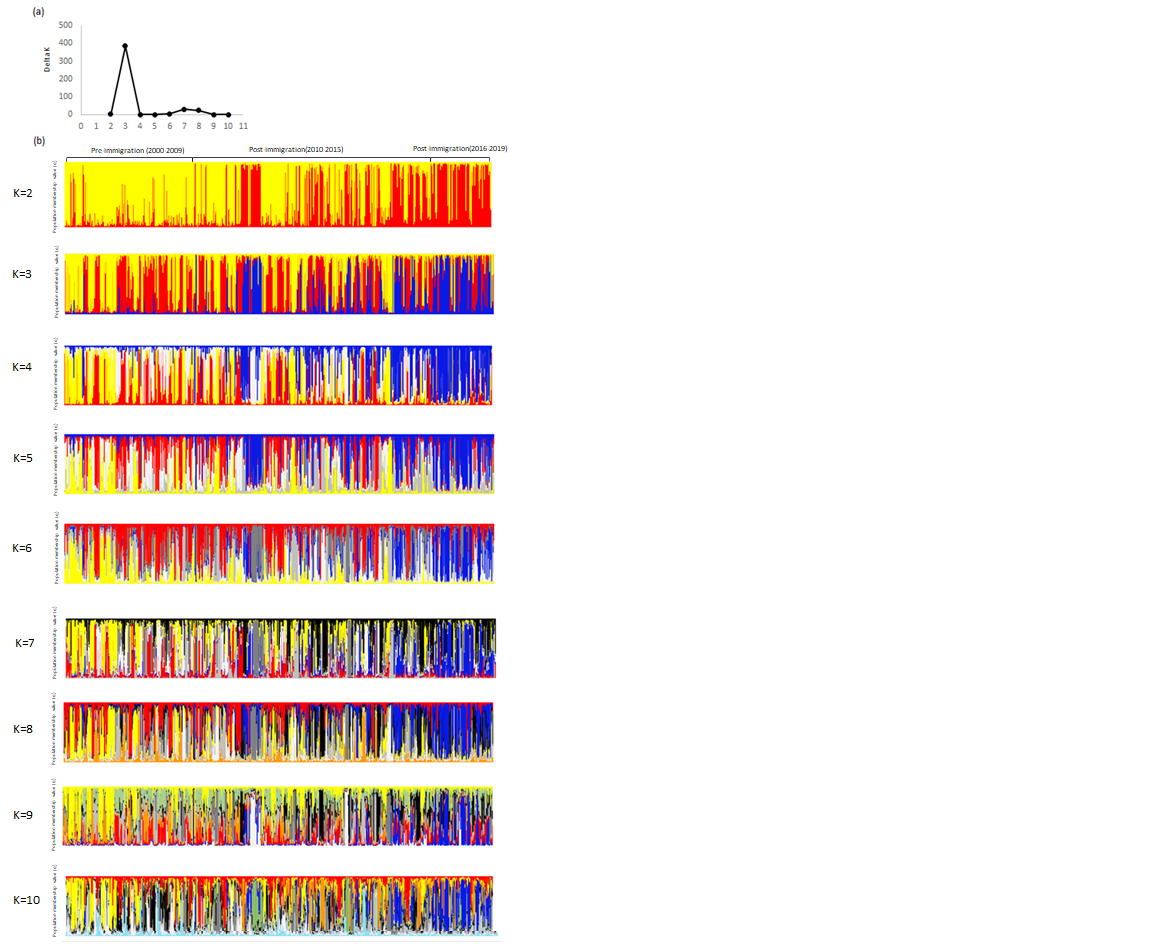


**Fig S2.** (a) Delta K for K=1-10 with three consecutive repeats, b) STRUCTURE bar plots for K=2-10 without prior population info
